# Assessment of salivary microbiota profile as a potential diagnostic tool for pediatric celiac disease

**DOI:** 10.1038/s41598-024-67677-4

**Published:** 2024-07-19

**Authors:** Asal Noruzpour, Fahimeh Sadat Gholam-Mostafaei, Mehdi Azizmohammad Looha, Hossein Dabiri, Shokoufeh Ahmadipour, Pejman Rouhani, Carolina Ciacci, Mohammad Rostami-Nejad

**Affiliations:** 1 Department of Microbiology, School of Medicine, Shahid Beheshti University of Medial Science, Tehran, Iran; 2 Gastroenterology and Liver Diseases Research Center, Research Institute for Gastroenterology and Liver Diseases, Shahid Beheshti University of Medical Sciences, Tehran, Iran; 3 Basic and Molecular Epidemiology of Gastrointestinal Disorders Research Center, Research Institute for Gastroenterology and Liver Diseases, Shahid Beheshti University of Medical Sciences, Tehran, Iran; 4 Pediatric Gastroenterologist, Hepatitis Research Center, Lorestan University of Medical Sciences, Khorramabad, Iran; 5 Department of Pediatric Gastroenterology and Hepatology, Mofid Children’s Hospital, Shahid Beheshti University of Medical Sciences, Tehran, Iran; 6 Department of Medicine and Surgery, University of Salerno, Salerno, Italy; 7 Celiac Disease and Gluten Related Disorders Research Center, Research Institute for Gastroenterology and Liver Diseases, Shahid Beheshti University of Medical Sciences, Tehran, Iran

**Keywords:** Celiac disease, Salivary microbiota, Oral dysbiosis, Pediatric patients, Microbiology, Gastrointestinal diseases

## Abstract

The association between oral dysbiosis and celiac disease (CD) remains poorly understood, as does the impact of CD-associated dysbiosis on disease development or exacerbation. This study aims to investigate alterations in salivary microbial composition among children with CD. In this cross-sectional study, saliva samples from 12 children with active CD (A-CD group), 14 children with CD on a gluten-free diet (GFD), and 10 healthy control (HC) children were analyzed using DNA sequencing targeting the 16S ribosomal RNA. Both patients in A-CD and GFD groups showed a significant increase (p = 0.0001) in the Bacteroidetes phylum, while the Actinobacteria phylum showed a significant decrease (p = 0.0001). Notably, the *Rothia* genus and *R.aeria* also demonstrated a significant decrease (p = 0.0001) within the both CD groups as compare to HC. Additionally, the control group displayed a significant increase (p = 0.006) in *R.mucilaginosa* species compared to both CD patient groups. Distinct bacterial strains were abundant in the saliva of patients with active CD, indicating a unique composition of the salivary microbiome in individuals with CD. These findings suggest that our approach to assessing salivary microbiota changes may contribute to developing noninvasive methods for diagnosing and treating CD.

## Introduction

Celiac disease is an autoimmune disorder characterized by small intestine inflammation, primarily affecting genetically predisposed individuals^1^. In these individuals, gluten consumption triggers an innate and adaptive immune responses, resulting in intestinal damage2^2^]. Recent studies have challenged the notion that celiac disease is solely due to individual gluten sensitivity and have proposed other environmental factors, such as microbial dysbiosis, may contribute to its pathogenesis^3–6^.

The oral microbiome, which comprises a diverse community of approximately 100 to 200 bacterial species such as *Firmicutes* spp., *Bacillus* spp., *Proteobacteria*, and *Actinomycetes*, is considered one of the most complex microbial communities in the human body^7^. However, the composition of the oral microbiota undergoes dynamic changes throughout different stages of life, health, and diseases^8^, influenced by factors such as dental procedures, diet, and medications^9^. Notably, the salivary microbial composition can impact the intestinal microbiota since it is continuously ingested. The role of the alimentary canal microbiota in celiac disease pathogenesis has gained attention. This is supported by evidence demonstrating the involvement of intestinal microbiota in processes such as intestinal differentiation, permeability, immunity, and tolerance^10–13^. Moreover, specific bacterial species, including *Rothia aeria, Pseudomonas aeruginosa, Lactobacillus* spp., and *Neisseria flavescens*^14,15^, have been implicated in influencing the immunogenicity of gluten peptides and inflammatory signals^16,17^. While a few studies have explored the salivary microbiome in children with celiac disease compared to duodenal and fecal microbiomes^18^, further investigation is warranted.

Diagnosing celiac disease typically involves invasive procedures such as intestinal biopsy, and alternative methods like genetic tests often lack definitive conclusions. Therefore, the development of non-invasive diagnostic and monitoring approaches is crucial. The assessment of oral microbiota has emerged as a potential tool due to its accessibility, prompt analysis, and cost-effectiveness^19^.

In this study, we aimed to investigate the composition of the salivary microbiome in children with active celiac disease and those on a gluten-free diet compared to healthy subjects and identify oral specific markers that may contribute to the development of novel diagnostics for pediatric CD in the future.

## Results

### Patients

This cross-sectional study examined of 36 children consisted of 12 A-CD children, 14 children under GFD and 10 healthy subjects (Table 1). In this study, no significant correlation was found among the three study groups in terms of children's weight and height (p = 0.203 and p = 0.658 respectively).

**Table 1:** Demographic data of participants.

Statistical population	Sample number	Gender		Age mean (range)	Height(cm) mean	Weight (Kg) mean
		Male(n) (%)	Female(n) (%)			
GFD-CD	14	5 (35.7%)	9 (64.2%)	8 (2–13)	123.86	24.70
HC	10	6 (60%)	4 (40%)	7.2 (2–14)	118.50	22.33
A-CD	12	2 (16.66)	10 (83.3%)	9.3 (3–14)	130.00	31.36

*GFD-CD* CD on a gluten-free diet, *HC* healthy control, *A-CD* active CD.

The most common clinical presentations in A-CD group were stomachache (91.66%), anemia (33.33%), weight loss (75%), constipation (57.14%), vomiting (25.21%).

Microbial analysis was performed by quantitative real-time PCR (RT-qPCR) assay targeting the 16S rRNA gene. In terms of clinical presentations at diagnosis, some strains were significantly related to specific symptom presentations of the disease; vomiting was significantly related to *Gammaproteobacteria* (p = 0.034) and *Rothia* area (p = 0.054), stomachache was more common in cases with higher abundance of Bacteroidetes phyla (p = 0.021), anemia was linked with *Streptococcus* (p = 0.039) and *Gammaproteobacteria* (p = 0.041), and there were significant relationship between constipation and *Gammaproteobacter* (p = 0.056).

Salivary microbiota analysis was conducted in all three groups, and the relative abundance of each bacterial taxa was calculated (Table 2).

**Table 2:** Relative abundance of the bacterial taxa studied in this work.

Microbiota taxa	A-CD	GFD-CD	HC	P-value*
*Actinobacteria*	3.99 ± 6.40	4.99 ± 6.97	8.08 ± 9.86	.001*
*Bactroidetes*	10.67 ± 14.50	8.63 ± 11.95	5.03 ± 11.38	.00001*
*Fusobacteria*	4.43 ± 5.88	4.37 ± 6.63	4.12 ± 5.73	.313
*Tenericutes*	.00 ± 2.24	.00 ± 2.74	.00 ± 2.46	.266
*Firmicutes*	8.98 ± 14.48	9.09 ± 13.31	9.57 ± 13.40	.205
*Alphaproteobacter*	3.75 ± 4.86	3.47 ± 5.26	3.32 ± 6.45	.301
*Betaproteobacter*	6.85 ± 8.00	3.93 ± 5.37	3.24 ± 4.71	.00001*
*Gamaproteobacter*	3.33 ± 4.92	3.23 ± 4.91	3.15 ± 5.49	.448
*Veillonella spp.*	.00 ± 5.57	.01 ± 2.54	.02 ± 1.56	.686
*Prevotella spp.*	2.35 ± 4.15	4.51 ± 6.97	2.92 ± 5.21	.001*
*Neisseria spp.*	5.39 ± 7.04	4.05 ± 5.71	2.38 ± 4.38	.00001*
*Gemella spp.*	2.83 ± 5.71	2.78 ± 5.52	2.13 ± 4.09	.151
*Clostridium*	2.71 ± 3.47	2.79 ± 3.98	3.47 ± 4.28	.683
*Streptococcus spp.*	4.71 ± 6.72	4.88 ± 6.94	3.49 ± 5.86	.041*
*Eubacterium s*	4.63 ± 5.21	4.56 ± 4.56	4.33 ± 5.28	.232
*Bifidobacterium spp.*	2.73 ± 4.52	2.67 ± 4.05	2.74 ± 4.24	.858
*Lactobacillus*	3.13 ± 4.87	3.60 ± 4.77	3.84 ± 5.20	.245
*Rothia spp.*	3.33 ± 4.64	3.48 ± 6.18	4.87 ± 7.39	.002*
*Rothia-dentocariosa*	1.13 ± 3.21	2.49 ± 3.41	2.07 ± 3.40	.274
*Rothia-mucilaginosa*	.00 ± 3.94	3.03 ± 4.61	2.82 ± 4.42	.006*
*Rothia-aeria*	1.47 ± 2.91	2.01 ± 3.54	2.58 ± 4.67	.00001*
*Granulicatella-adiacens*	2.10 ± 4.68	2.10 ± 3.93	2.11 ± 3.80	.898

### Microbial communities in healthy controls and pediatric CD patients

**Figure 1:**
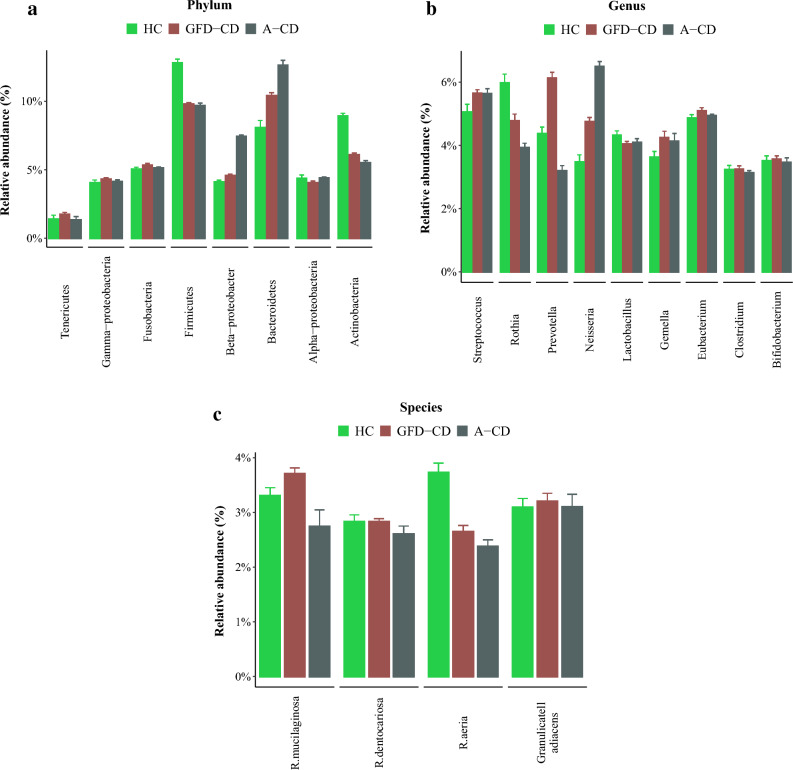
Bar-plot analysis of the relative abundance and distribution of each targeted oral microbiota in saliva samples of healthy controls, GFD-CD and active CD (A-CD). Saliva microbiota composition and alterations at the (**a**) phylum (**b**) genus and (**c**) spices level data are presented as mean ± SD.

**Figure 2:**
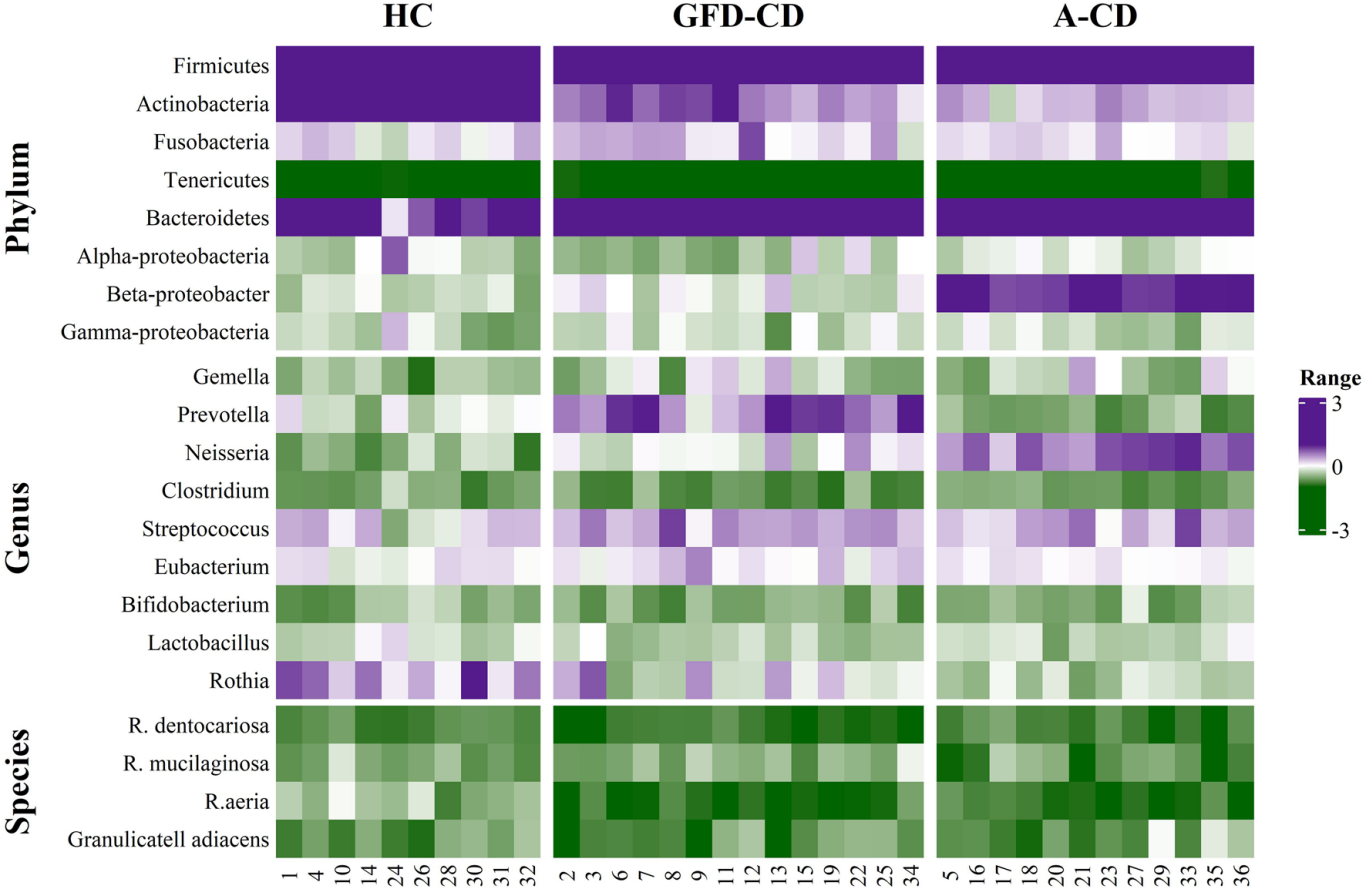
The relative percentage and changes of targeted oral microbiota communities in saliva samples of healthy controls, GFD-CD and active CD (A-CD) salivary microbiota. Salivary microbiota composition and alterations at the phylum, genus and spices level. Data are presented as mean ± SD. Each color corresponds to a type of microbiota included in this study.

There were significant differences observed at the phylum, genus, and species levels among the three study groups (Figs. 1 and 2). In both patient groups, the most abundant phylum was *Bacteroides* (p = 0.00001) (Fig. 1). In contrast, *Actinobacteria* and *Firmicutes* were the most abundant phyla in the healthy control (HC) group, and also *Actinobacteria* showing a significant increase in this group compared to the patients (p = 0.001). *Beta-proteobacteria* dominated in the A-CD group.

At the genus level, *Prevotella* and *Streptococcus* were the most abundant in the GFD-CD group, while *Neisseria* and *Streptococcus* were dominant in the A-CD group. Notably, there was a significant increase in the healthy control group for the genus *Rothia* (p = 0.002) (Fig. 2). The most prevalent species in the GFD-CD, A-CD, and HC groups were *Rothia mucilaginosa*, *Granolicatella adiacens*, and *Rothia aeria*, respectively. (p = 0.001) Furthermore, we observed significant increase in abundance of *R*.*aeria* (p = 0.00001) and *R.musilaginosa* (p = 0.006) in the healthy control group compared to both CD patient groups.

### Dissimilarity and principal coordinates analysis.

**Figure 3:**
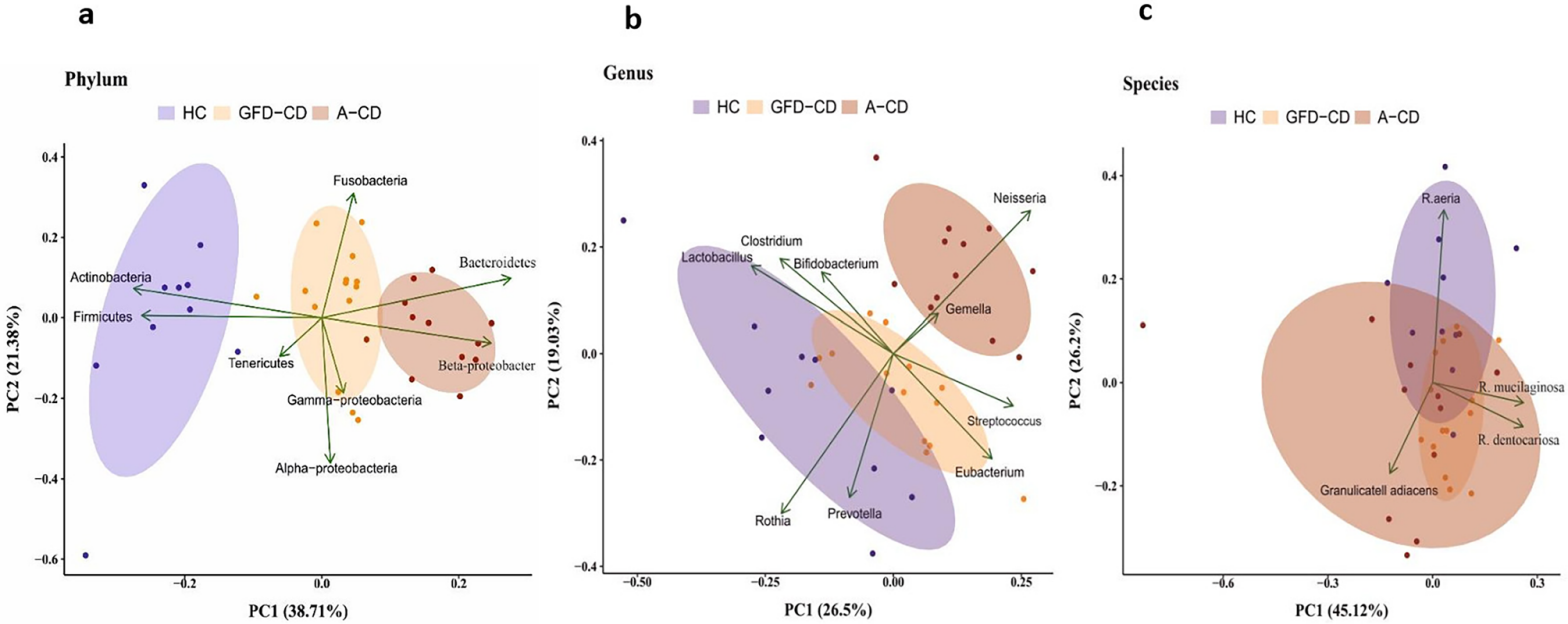
Bacterial community clustering and variations using principal component analysis (PCA) in saliva samples of HC, GFD-CD and A-CD saliva microbiota. (**a**) PCA of saliva microbiota clustering at the phylum level. (**b**) PCA of salivary microbiota clustering at the genus level. (**c**) PCA of saliva microbiota clustering at the spices level. Percentage values in parentheses next to Dim1 and Dim2 represent the percentage of variance explained by each component. Arrows show the contribution of each type of microbiota on Dim1 and Dim2. Each data point denotes an individual patient, colored based on their group.

Descriptive statistics revealed that the microbial community in individuals following a gluten-free diet exhibited a notable shift towards the healthy control group. Additionally, the composition of the bacterial community showed a similar shift towards the healthy control group when compared to children in the A-CD group. Furthermore, Fig. 3 provides a schematic representation of the PCA analysis highlighting the overall differences in microbial composition among the three groups.

## Discussion

The composition of salivary microbiota shares similarities with other parts of the digestive system, such as the stomach and duodenum, in terms of bacterial species^20^. Given that the mouth serves as the initial stage of digestion, the oral bacterial composition continually influences the microbial composition of the intestines^8^. However, the research on the relationship between salivary microbiota and various diseases remains insufficient compared to the extensive study of microbiota in other sections of the digestive system.

Regarding children with celiac disease (CD), the presence of oral dysbiosis has not yet been definitively established. Furthermore, there is limited understanding of the underlying mechanisms responsible for changes in oral microbiota among children with CD. Our findings revealed marked differences in the oral microbiome between CD patients with active CD and on a GFD and the control group.

Our results indicate that children with CD exhibit salivary dysbiosis characterized by notable shifts in microbial composition. Specifically, the abundance of Firmicutes and Actinobacteria phyla was considerably lower in children with active CD and those following a GFD compared to healthy children. This significant disparity suggests that analyzing oral microbial composition could potentially serve as an early screening and diagnostic tool for children with CD.

Existing literature confirms disparities in the microbiomes of individuals with CD children when compared to healthy controls, including reduced abundance of *Actinobacteria*and *Firmicutes* and increase abundance of Bacteroidetes^21^. Interestingly, specific species within the *Firmicutes* phylum, such as *Lactobacillus* and *Bacillus cereus*, which are known to impact gluten metabolism, were undetectable in the microbiomes of CD patients^22,23^. Moreover, low levels of *Lactobacilli* have been observed in both adults and children with active CD. Additionally, intestinal microbial levels of *Firmicutes*, *Lactobacillus*, and *Actinobacteria* have been found to be lower in individuals with active CD when compared to healthy individuals, suggesting a correlation with gluten metabolism differences^24,25^.

The microbiota may not only modulate immune responses but also contribute to CD by aiding in the elimination of harmful gluten peptides and participating in gluten detoxification. For instance, a cocktail of oral bacteria, or their isolated enzymes might play a minor protective role against deleterious gliadin peptides^26^. It is postulated that CD-associated bacteria could influence intracellular trafficking by evading lysosomal degradation and triggering gliadin-independent stress pathways or interfering with toxic gliadin peptide pathways. Nevertheless, it remains unclear whether the bacterial species associated with CD actively contribute to disease pathogenesis or are merely an outcome of the condition^27^.

Our findings indicated that there was a significant decrease in *Actinobacteria* in this group of patients, implying a possible association between genera from this phylum, such as *Rothia*, and gluten breakdown.

In the future, the analysis of saliva microbiota in pediatric patients holds great potential for early CD diagnosis and timely interventions. Our study demonstrates promising evidence that saliva analysis in children can predict future oral and systemic diseases. However, considering the limited research in this field, our findings address current gaps and provide substantial evidence supporting the notion that oral microbiota analysis could be invaluable in predicting CD and potentially other diseases.

Several limitations should be acknowledged in our study. At first low sample size is the main limitation of this study. Then CD children under GFD included in the present study adhered to specific diet and the impact of different gluten-free diets on oral microbiota colonization remains unclear, one study reported distinct compositions of the oral microbiome associated with different gluten-free diets. Additionally, we did not examine or incorporate factors such as oral hygiene and dental health, which could have influenced the abundance of different bacterial genera. It is worth noting that previous studies have predominantly been conducted in developed countries with higher standards of oral and dental hygiene^28^. In contrast, our study took place at a teaching hospital in a low-income developing country. Furthermore, due to time and budget constraints, our oral examination was limited to visible lesions or marks from prior surgeries. This limited scope might be considered a drawback of the study, and future research should encompass a comprehensive oral and dental examination given the dynamic nature of oral microbial composition.

## Conclusion

In conclusion, this study provides evidence that celiac disease (CD) can lead to dysbiosis in the oral microbiota, characterized by an abundance of *Bacteroidetes* strains. These findings suggest that the specific CD-associated microbiota may contribute to the development of the disease. Furthermore, these results have implications for the screening and diagnosis of CD in children. However, it is important to note that a limitation of this study is the lack of information on the oral and dental health status of the children. Future research directions could involve investigating enzymes involved in gluten degradation within the bread and grain production industry or exploring the potential of next-generation probiotics.

## Methods

### Study population

A total of 36 eligible Iranian children aged 2 to 14 years (24 females and 12 males) were invited to participate in the study during 2021–2022. Among them, 12 were active celiac disease (mean age ± SD = 9.3 ± 4) who had not been on a gluten-free diet (referred to as A-CD group), 14 CD children who were on a gluten-free diet (mean age ± SD = 8 ± 5) for at least twelve months (referred to as GFD-CD group), and 10 healthy control subjects (mean age ± SD = 7.2 ± 3). All relevant medical and laboratory data (including serological results (tTG IgA), pathological reports (Marsh I-III) and clinical presentations) were collected and recorded in a single clinical history file. The patient groups were diagnosed using serological and histological tests, while the healthy controls had no gastrointestinal or CD-related autoimmune diseases, food intolerances or allergies, active immunological diseases, or were not taking immune system-related medications. Participants who met any of these criteria were excluded from the study.

### Exclusion criteria

The following individuals were excluded from the study: those with known gastrointestinal and immunological disorders or food intolerances or allergies (excluding gluten intolerance), IgA deficiency, recent use of antibiotics, proton pump inhibitors, antiviral or corticosteroids, or consumption of probiotics within the two months prior to sampling. Additionally, individuals with a history of gum surgery or systemic use of immunosuppressants or antibiotics were excluded within the four weeks before the study. GFD-CD group were under strict diet and those who reported any symptoms were excluded from study.

### Saliva sample collection and DNA extraction

Saliva samples were collected by trained personnel after oral evaluation. Sampling was performed in the morning, two hours after enamel brushing. Participants were instructed not to eat or drink anything (except water) before sampling. Saliva samples were collected by spitting into a sterile collection kit (Oragene-DNA610) and immediately frozen at −20 °C. Children with confirmed active dental or periodontal lesions were excluded from the study. Prior to sample collection, participants were required to refrain from eating, drinking, tooth brushing, and rinsing their mouth (except water) for at least two hours. After the two-hour fasting period, saliva samples were collected using the sterile collection kit mentioned above. The frozen samples were stored at −20 °C until further analysis. DNA extraction from the saliva samples was performed according to the manufacturer's instructions using the Qiamp DNA mini kit (Qiagen Retsch GmbH, Hannover, Germany). The concentration and purity of the extracted DNA were assessed using the Nanodrop ND-2000 Spectrophotometer (DeNovix Inc., USA).

### Saliva microbiota analysis by quantitative real-time PCR

The microbial composition in the saliva samples was analyzed using quantitative real-time PCR (qRT-PCR) targeting the 16S rRNA gene. SYBR Green Chemistry was used, and universal and group-specific primers based on bacterial 16S rRNA sequences (Table 3) were utilized. Each PCR reaction was conducted in a final volume of 20 μl, consisting of SYBR green PCR master mix (Ampliqon, Odense, Denmark), 0.4 μl of forward and reverse primers, and 1 μl of 100 ng DNA template. The amplification parameters were as follows: initial denaturation at 95 °C for 10 min, followed by 40 cycles of denaturation at 95 °C for 20 s, annealing at the optimum temperature for each primer pair for 30 s, and extension at 72 °C for 20 s. The amplification was repeated three times using a Rotor-Gene® Q real-time PCR system (Qiagen, Germany). Melting curve analysis was performed to confirm amplification specificity by increasing the temperature from 60 to 95 °C in regular steps of 0.5 °C for 5 s. The relative abundance of each taxon in the three groups was calculated based on the ratio of the 16S rRNA copy number of specific bacteria to the total 16S rRNA copy number of universal bacteria. The average Ct values obtained from each primer pair were converted to percentages using the following formula:

$$ x = \frac{{\left( {eff.uni} \right)^{ct\,uni} }}{{\left( {eff.spec} \right)^{ct\,spect} }} \times 100 $$

**Table 3:** Primer sequences used for RT-PCR.

Target taxon	Primer name	Primer sequence	Amplicon length	References
*Eubacteria*	UniF340	AGMGTTYGATYMTGGCTCAG	∼ 200	Moraes JGd, et al.^29^
	UniR514	GCTGCCTCCCGTAGGAGT		
*Bacteroidetes*	Bac960-F	GTTTAATTCGATGATACGCG	∼ 137	Matsuki T, et al.^30^
	Bac1100-R	TTAAGCCGACACCTCACG		
Firmicute	Firm934-F	GGAGYATGTGGTTTAATTCGAAGCA	∼ 129	Matsuki T, et al.^30^
	Firm1060-R	AGCTGACGACAACCATGCAC		
*Actinobacteria*	Actino-F	GCGKCCTATCAGCTTGTTGGTG	∼ 333	Hermann-Bank ML, et al.^31^
	Actino-R	CCGCCTACGAGCYCTTTACGC		
*Fusobacteria*	Fuso-F	GATCCAGCAATTCTGTGTG	∼ 290	Hermann-Bank ML, et al.^31^
	Fuso-R	CGAATTTCACCTCTACACTTG		
Tenericutes	Ten662-F	ATGTGTAGCGGTAAAATGCGTAA	∼ 219	Yang Y-W.^32^
	Ten862-R	CMTACTTGCGTACGTACTACT		
Alpha-proteobacteria	α682-F	CGAGTGTAGAGGTGAAATTC	∼ 305	De Gregoris TB et al.^33^
	α968-R	GGTAAGGTTCTGCGCGTT		
Beta-proteobacteria	Beta979-F	AACGCGAAAAACCTTACCTAC	∼ 175	Yang Y-W.^32^
	Beta1130-R	GCCCTTTCGTAGCAACTA		
Gamma-proteobacteria	Gamma395-F	CMATGCCGCGTGTGTGAA	∼ 498	De Gregoris TB et al.^33^
	Gamma871-R	ACTCCCCAGGCGGTCDACTTA		
*Streptococcus spp.*	Str1-F	GTACAGTTGCTTCAGGACGT	∼ 195	Hermann-Bank ML, et al.^31^
	Str2-R	GTTCGATTTCRTCACGTTG		
*Prevotella spp.*	g-Prevo-F	CACRGTAAACGATGGATGC	∼ 507	Gholam-mostafaei F,et al.^34^
	g-Prevo-R	TTGCAGACCCCAGTCCGAAC		
*Bifidobacterium spp.*	Bifid-F	GGGATGCTGGTGTGGAAGAG	∼ 200	Wang I-K et al.^35^
	Bifid-R	TGCTCGCGTCCACTATCCAG		
*Veillonella spp.*	Veil-F-RinttiläR	AYCAACCTGCCCTTCAGA	∼ 343	Rinttilä T.^36^
	Veil-R-Rinttilä	CGTCCCGATTAACAGAGCTT		
*Gemella spp.*	GemF	AAAGCTCTGTTGTTAGGGAA	∼ 98	Sun, B., et al.^37^
	GemR	P-E- GGTGGCTTTCTGGTTAGGTA		
*Clostridium coccoides group*	g-Ccoc-F	AAATGACGGTACCTGACTA	∼ 438	Matsuki et al.^38^
	g-Ccoc-R	CTTTGAGTTTCATTCTTGCGA		
*Eubacterium*	Eub F	P-E- GATACCCTGGTAGTCCACGC	*∼*146	Sun, B., et al.^37^
	Eub R	Q-E- CTCCCCAGGTGGAATACTTA		
*Neisseria spp.*	F	CTGGCGCGGTATGGTCGGTT	*∼* 103	Esposito MV.^39^
	R	GCCGACGTTGGAAGTGGTAAAG		
*Rothia spp.*	Rothia_1F	GGGACATTCCACGTTTTCCG	*∼* 322	Lim YW.^40^
	Rothia_1R	TCCTATGAGTCCCCACCATT		
*Rothia aeria*	F	GCATTAGATCGCGTCAGAG	*∼* 143	Tsuzukibashi O.^41^
	R	GGCCGAACCGCTGGCAACA		
*Rothia mucilaginosa*	F	GCCTAGCTTGCTAGGTGGAT	*∼* 163	This study
	R	GAGCCCATCTATAACCACTAC		
*Rothia dentocariosa*	F	GCCTAGCTTGCTAGGTGGAT	*∼* 127	This study
	R	AACACCCCATGCGGAGATTG		
*Granulicatella adiacens*	F	CAAGCTTCTGCTGATGGATGGA	*∼* 89	Alkharaan H.^42^
	R	CTCAGGTCGGCTATGCATCAC		

The term “eff.uni” was used to represent the calculated performance of the generic primers, with a value of 2 indicating a hundred percent efficiency and 1 indicating zero percent efficiency. Similarly, “eff.spec” referred to the performance of the taxon-specific primers. The threshold cycles registered by the thermal cycler were denoted as “ct univ” and “ct spec.” The variable ‘x’ represented the percentage of taxon-specific 16 s copy number present in the sample.

### Statistical analysis

Spearman’s correlation analysis was employed for nonparametric data, while Student's t-test and Mann–Whitney test were used for parametric data analysis. PCA (principal component analysis) plots were generated using the FactoMineR and Factoextra packages within the open-source statistical program R version 3.6.1 (R Core Team, Vienna, Austria). The relative abundance of microbiota was visualized using GraphPad Prism software version 8.3.0 (GraphPad Software, San Diego, CA, USA). Statistical significance was considered when *p < 0.05, **p < 0.01, and ***p < 0.001.

Descriptive statistics were reported as mean ± standard deviation (SD) or median (interquartile range [IQR]) for numeric variables. Categorical variables were presented as frequency (percentage).

Barplots were used to visually represent the composition of the microbiota at the levels of Genus, Species, and Phylum. Raw abundance counts for each participant were initially obtained for the microbial taxonomic levels (Genus, Phylum, and Species). Prior to analysis, appropriate data preprocessing steps, including normalization and transformation, were applied to ensure data comparability and reliability. The Heatmap function from the Complex Heatmap package was employed to visualize the microbial composition and highlight group differences. Each row in the heatmap represented a specific microbiota (Genus, Phylum, or Species), while each column represented a participant from their respective groups.

To classify the microbiota into the HC, GFD-CD, and A-CD groups at the Genus, Phylum, and Species levels, Principal Component Analysis (PCA) was performed. This analysis aimed to identify patterns and potential differences in the composition of the microbiota between the groups.

All statistical analyses were conducted using the R programming language (version 4.2.1) and relevant packages, including ComplexHeatmap and ggplot2, among others, as appropriate. Statistical significance was defined as p-values less than 0.05.

### Study design

This cross-sectional study was conducted at the Celiac Disease and Gluten Related Disorders Research Center, Shahid Beheshti University of Medical Sciences, Tehran, Iran between March 2021 and December 2022. Ethics Committee of Shahid Beheshti University of Medical Sciences (Project No. IR.SBMU.RIGLD.REC.1398.047) approved the research, and confirmed that all research was performed in accordance with relevant guidelines/regulations, and also confirmed that informed consent was obtained from all participants and/or their legal guardians.

## Data Availability

The datasets used and/or analyzed during the current study available from the corresponding author on reasonable request.
